# Characterization of the gibberellic oxidase gene *SdGA20ox1* in *Sophora davidii* (Franch.) skeels and interaction protein screening

**DOI:** 10.3389/fpls.2024.1478854

**Published:** 2024-10-16

**Authors:** Lili Zhao, Wenhui Xie, Lei Huang, Sisi Long, Puchang Wang

**Affiliations:** ^1^ College of Animal Science, Guizhou University, Guiyang, China; ^2^ School of Life Sciences, Guizhou Normal University, Guiyang, China

**Keywords:** Sophora davidii, gibberellin 20-oxidases, gibberellins, plant height, interacting protein

## Abstract

Gibberellin 20-oxidases (*GA20oxs*) are multifunctional enzymes involved in regulating gibberellin (GA) biosynthesis and controlling plant growth. We identified and characterized the GA20ox1 gene in a plant height mutant of *Sophora davidii*, referred to as *SdGA20ox1*. This gene was expressed in root, stem, and leaf tissues of the adult *S. davidii* plant height mutant, with the highest expression observed in the stem. The expression of *SdGA20ox1* was regulated by various exogenous hormones. Overexpression of *SdGA20ox1* in Arabidopsis resulted in significant elongation of hypocotyl and root length in seedlings, earlier flowering, smaller leaves, reduced leaf chlorophyll content, lighter leaf color, a significant increase in adult plant height, and other phenotypes. Additionally, transgenic plants exhibited a substantial increase in biologically active endogenous GAs (GA1, GA3, and GA4) content, indicating that overexpression of *SdGA20ox1* accelerates plant growth and development. Using a yeast two-hybrid (Y2H) screen, we identified two SdGA20ox1-interacting proteins: the ethylene receptor *EIN4* (11430582) and the *rbcS* (11416005) protein. These interactions suggest a potential regulatory mechanism for *S. davidii* growth. Our findings provide new insights into the role of *SdGA20ox1* and its interacting proteins in regulating the growth and development of *S. davidii*.

## 1 Introduction

Plant height is a crucial component of plant structure, significantly impacting crop yield and quality (Wang et al., 2017). Many genes associated with plant height are closely linked to phytohormone synthesis, transportation, and signal transduction (Wang and Li, 2008; Ku et al., 2015). For example, the semidwarf and high-yield varieties of the “Green Revolution” resulted from changes in gibberellin (GA) biosynthesis and signaling pathways (Hedden, 2003). Gibberellin is a plant hormone that affects plant growth and development, playing a role in seed germination, stem elongation, leaf growth, and flowering (Yamaguchi, 2008). Gibberellin 20-oxidases (*GA20oxs*) are critical regulatory enzymes in the GA synthesis pathway, acting as soluble dioxygenases that regulate the synthesis of biologically active GA4 and GA1 in plants (Phillips et al., 1995). Many *GA20oxs* genes have been isolated and characterized in Arabidopsis (*Arabidopsis thaliana*), tobacco (*Nicotiana tabacum*), and other plants. For instance, overexpression of *AtGA20ox1* in *Arabidopsis thaliana* led to earlier flowering and increased plant height (Coles et al., 1999). Similarly, overexpression of *CrGA20ox1* promoted increased height in *Camellia reticulata* compared to wild-type plants (Wang et al., 2018), and overexpression of *StGA20ox1* in *Solanum tuberosum* resulted in increased plant height and a requirement for longer daylight to produce tubers (Carrera et al., 2000). In contrast, overexpression of *PdGA20ox1* in the woody plant *Pinus densiflora* significantly enhanced stem growth and increased stem weight (Jeon et al., 2016).

*Sophora davidii* (Franch.) Skeels (2n = 18) is a typical deciduous shrub or small tree found in the mountains of Guizhou Province, Southwest China (Zhao et al., 2022a). In recent decades, stone desertification in Southwest China has severely threatened the local ecology (Ding et al., 2020). *S. davidii*, with its deep and well-developed root system, has been widely used for vegetation restoration to repair damaged ecosystems (Zhao et al., 2022c, Zhao et al., 2024). The stems and leaves of *S. davidii* are tender, tasty, and rich in protein, vitamins, minerals, amino acids, alkaloids, and flavonoids, making it a high-quality local fodder with medicinal value (Zhao et al., 2022b). However, adult *S. davidii* plants can reach up to 2 meters in height, which hinders the normal feeding of shorter livestock such as goats. Conventional breeding methods to develop new dwarf cultivars are extremely difficult and time-consuming. However, by analyzing the functions of genes regulating the height of *S. davidii* plants, genome editing technologies can be used to accelerate the breeding of new semi-dwarf or dwarf phenotypes of *S. davidii*, promoting the development of local ecological livestock farming.

To date, no studies have investigated the mechanism of *S. davidii* seedling height growth. Our research team previously conducted preliminary studies on *S. davidii* plant height mutants obtained through mutagenesis and speculated that the *GA20ox1* gene plays an important role in plant height regulation (Xie et al., 2023; Zhao et al., 2022b). In this study, we isolated *SdGA20ox1* from *S. davidii* plant height mutants, analyzed its encoded protein sequence, and introduced it into Arabidopsis to validate its function. We constructed a yeast nuclear library and screened for proteins interacting with *SdGA20ox1* using yeast two-hybrid (Y2H) and bimolecular fluorescence complementation (BiFC) techniques. To our knowledge, this is the first report on the isolation of the GA20-oxidase gene from *S. davidii*. The research results reveal the function of the *SdGA20ox1* gene, enriching the knowledge of *GA20ox1* and providing material support for the genetic engineering of plant height mutant varieties.

## 2 Materials and methods

### 2.1 Plant materials and growth conditions

Wild-type (WT) *Sophora davidii* and radiation-induced plant height mutants grown in Guiyang, Guizhou, China, were used in this study, similar to previous studies (Zhao et al., 2022b). These plants were grown under natural light conditions with regular irrigation. *Arabidopsis thaliana* ecotype Columbia (Col-0) was used as the WT and grown alongside the second generation (T2) of pure transgenic Arabidopsis in a growth chamber set at 22 ± 2°C with 70% humidity and 1200 Lux light intensity, following a 16-hour daytime and 8-hour darkness cycle. For sampling, *S. davidii* and Arabidopsis samples were immediately immersed in liquid nitrogen and stored at -80°C.

### 2.2 Hormone treatments

Three-month-old *S. davidii* plant height mutants with similar growth status were selected for various treatments. Hormone treatments included 10 μM Gibberellin A3 (GA3, CAS: 77-06-5), 5 μM brassinolide (BR, CAS: 72962-43-7), 10 μM indole-3-acetic acid (IAA, CAS: 87-51-4), 10 μM uniconazole (CAS: 83657-22-1), and 10 μM Ethephon (substitute for ethylene, ETH, CAS: 16672-87-0) (Xie et al., 2023). All hormone solutions were first dissolved in a small amount of ethanol and then diluted with distilled water to the appropriate concentration. The hormones were sprayed on the plants until the solution started to run off the leaves. Stems from the various treatments were sampled at the indicated time points (0, 3, 6, 9, 12, 24, 36, 48 hours) for qRT-PCR analysis.

### 2.3 Quantitative real-time PCR

Total RNA was extracted from plant tissues as described previously (Zhao et al., 2022a). Reverse transcription was performed using the HiFiScript cDNA synthesis kit (Cwbiotech), and gene-specific primers were synthesized using the PerlPrimer program. qRT-PCR was performed on an Applied Biosystems CFX ConnectTM real-time PCR detection system using UltraSYBR^®^ Mixture (Low ROX). The qRT-PCR thermal cycling parameters were: 95°C for 10 minutes followed by 40 cycles of 95°C for 15 seconds and 60°C for 1 minute, in 20 μL volumes. Expression data were analyzed using the 2-ΔΔCt method. Each reaction was performed with three independent biological replicates and three technical replicates per sample. All primers used for qRT-PCR are listed in **Supplementary Table S1**.

### 2.4 Cloning of *SdGA20ox1*

Total RNA was extracted from *S. davidii* as described in Zhao et al. (2022b), and the coding region of *SdGA20ox1* was amplified according to the manufacturer’s instructions based on the transcriptomic data previously obtained in our laboratory. The PCR products were subsequently cloned into the pGEM-T vector (Promega Corporation, Madison, WI, USA) and sequenced. Specific primer information for gene cloning is listed in **Supplementary Table S1**.. The 40 μL RT-PCR solution contained 5.0 μL of 2× Taq PCR Master Mix (Tian-gen, China), 1 μL forward and 1 μL reverse primers, 3.0 μL cDNA, and 15.0 μL deionized water. RT-PCR was performed with 30 cycles of 95°C for 30 seconds, 58°C for 30 seconds, and 72°C for 30 seconds, followed by 72°C for 10 minutes. The PCR product was then recovered and ligated into the pEASY-Blunt cloning vector (TransGen, Beijing, China), and sequenced by Sangon Biotech (Shanghai, China).

### 2.5 Sequences and phylogenetic analyses of *SdGA20ox1*

Amino acid sequences were obtained from the National Center for Biotechnology Information (http://www.ncbi.nlm.nih.gov), with the gene name *SdGA20ox1* and GenBank accession number ON229923. MEGA 11 software (https://www.megasoftware.net/megamac.php) and the online tool Evolview (https://www.evolgenius.info) were used to create phylogenetic trees (Subramanian et al., 2019). A neighbor-joining phylogenetic tree was generated using MEGA 11 with the Poisson model, gamma-distributed rates, and 1,000 bootstrap replicates.

### 2.6 Construction of plant overexpression vectors and transformation of *Arabidopsis thaliana*

The BamHI (5’) and PstI (3’) restriction sites were added to the ends of the *SdGA20ox1* CDS fragment, respectively, and the PCR products were cloned into the pHB vector and sequenced. The overexpression vector plasmid was transformed into Agrobacterium GV3101, and positive transgenic plants were identified with 50 μg/mL of kanamycin. The Agrobacterium strain carrying the pHB::SdGA20ox1 vector was transformed into Arabidopsis using the flower dip method (Clough and Bent, 1998). PCR primer sequences for transgenic plants are shown in **Supplementary Table S1**.. The transgenic Arabidopsis was screened for resistance using 50 μmol/L glufosinate until stable genetically pure T2 generations (OE2, OE5, and OE7) were obtained for subsequent experiments.

### 2.7 Plant phenotypic characterization

Wild-type and transgenic plants were grown under identical conditions, and seeds were collected simultaneously. Seed germination tests were conducted as described by Yan et al. (2017). Thirty seeds were sown on half-strength MS solid medium and incubated in the dark at 4°C for 4 days before being transferred to a growth chamber at 22°C. Seeds that completed germination were counted every 24 hours. The experiment was repeated three times. For seedling hypocotyl and root length determination, seeds were sown on half-strength MS solid medium. Plates were refrigerated at 4°C in the dark for 4 days, then moved to 22°C with a 16-hour light/8-hour dark cycle. On day 7, the hypocotyl lengths of approximately 10 seedlings were measured. On day 14, the root lengths of about 10 seedlings were measured. Arabidopsis seedlings grown on half-strength medium for 7 days were transplanted to pots for 45 days, and morphological characteristics (leaf length, plant height) were measured during the growing season.

### 2.8 Measurement of chlorophyll content

One gram of leaf tissue from 45-day-old Arabidopsis and 3-month-old *S. davidii* plants was collected and ground to a powder in liquid nitrogen. The powdered leaves were placed in 10 ml of extraction solution (95% ethanol: 80% acetone = 1:1 by volume), mixed, and stored overnight at 4°C in the dark. Total chlorophyll (Chl) was measured according to the method described by Yang et al. (2020), and each measurement was repeated three times.

### 2.9 Quantification of endogenous GA

Samples of wild-type and transgenic Arabidopsis were collected and weighed for further analysis. Young stems (from stem tip to node) were collected for GA content determination. The assay was performed as described previously by Chen et al. (2012), with three independent biological replicates and three technical replicates measured for each sample.

### 2.10 Subcellular localization and bimolecular fluorescence complementation assay

To study the subcellular localization of *SdGA20ox1*, specific primers with the enzymatic cut site BsaI/Eco31I were designed. The pBWA(V)HS-*SdGA20ox1*-Glosgfp vector was constructed, transformed into Escherichia coli (DH5α), and then transformed into tobacco leaves. The fluorescence signal was observed using confocal laser scanning microscopy (CLSM, Leica TCS SP2) with excitation and emission light at 511 nm and 525 nm, respectively. For the bimolecular fluorescence complementation (BiFC) assay, full-length *SdGA20ox1*, 11430582, and 11416005 genes were amplified using primers (**Supplementary Table S1**), cloned into the PpSm35s vector (Engler et al., 2008), and transiently transformed into the lower epidermis of tobacco leaves. After 48 hours of transfection, interactions were observed using CLSM with excitation at 511 nm and emission at 525 nm.

### 2.11 Yeast two-hybrid assay and retrieval of interacting proteins

To prevent self-activation of *SdGA20ox1*, a self-activation assay was performed. The decoy plasmid transformed recipient strain AH109 was cotransformed with pGBKT7-SdGA20ox1 and pGBKT7 using Saccharomyces cerevisiae. The transformed AH109 strain was plated on SD/-Trp plates deficient in tryptophan. Six randomly selected spots were verified by PCR with vector primers of pGBKT7. Three cloning sites were randomly selected and applied to culture medium deficient in tryptophan, histidine, and adenine, supplemented with X-α-Gal blue-displaying substrate (5-Bromo-4-chloro-3-indolyl-alpha-D-galactopyranoside), namely SD/-Trp, SD/-Trp/-His, SD/-Trp/-His/-Ade, and SD/-Trp/-His/-Ade+X-α-gal plates. Plates were incubated at 30°C for 3-5 days. pGBKT7 was used as the negative control, and the diameter and color of the colonies were observed and recorded.

To identify candidate interacting proteins of *SdGA20ox1*, a cDNA library of *S. davidii* mutants was constructed, and transformed cells were plated on SD/-Trp plates and cultured. Cells were then transferred to YPDA and cultured for 4–5 hours. Twenty microliters of culture were diluted and plated on SD/-TLH plates, incubated at 30°C for 3-7 days. Single colonies were picked and transferred to SD/-TLH plates for 3-5 days. Positive yeast clones were obtained by screening on SD/-TLH plates. Positive clones were amplified from yeast cells and sequenced. The positive clones were diluted and plated on SD/-TL, SD/-TLH, SD/-TLHA, and SD/-TLHA/+X-α-gal, respectively, and incubated at 30°C for 3 days. pGBKT7-p53 and pGADT7-largeT were co-transformed as positive controls, and pGBKT7-laminC and pGADT7-largeT were cotransformed as negative controls.

### 2.12 Statistical analysis

All experiments were performed at least three times. Error bars in each graph indicate the mean values ± SE of three replicates. Statistical significance between control and treatment groups was determined using Tukey’s test and Student’s t-test in SPSS Statistics software version 19 (IBM, USA). Different lowercase letters indicate significant differences (P < 0.05), with ** indicating *P* < 0.01 and * indicating *P* < 0.05.

## 3 Results

### 3.1 Cloning and sequence analysis of *SdGA20ox1*

**Figure 1:**
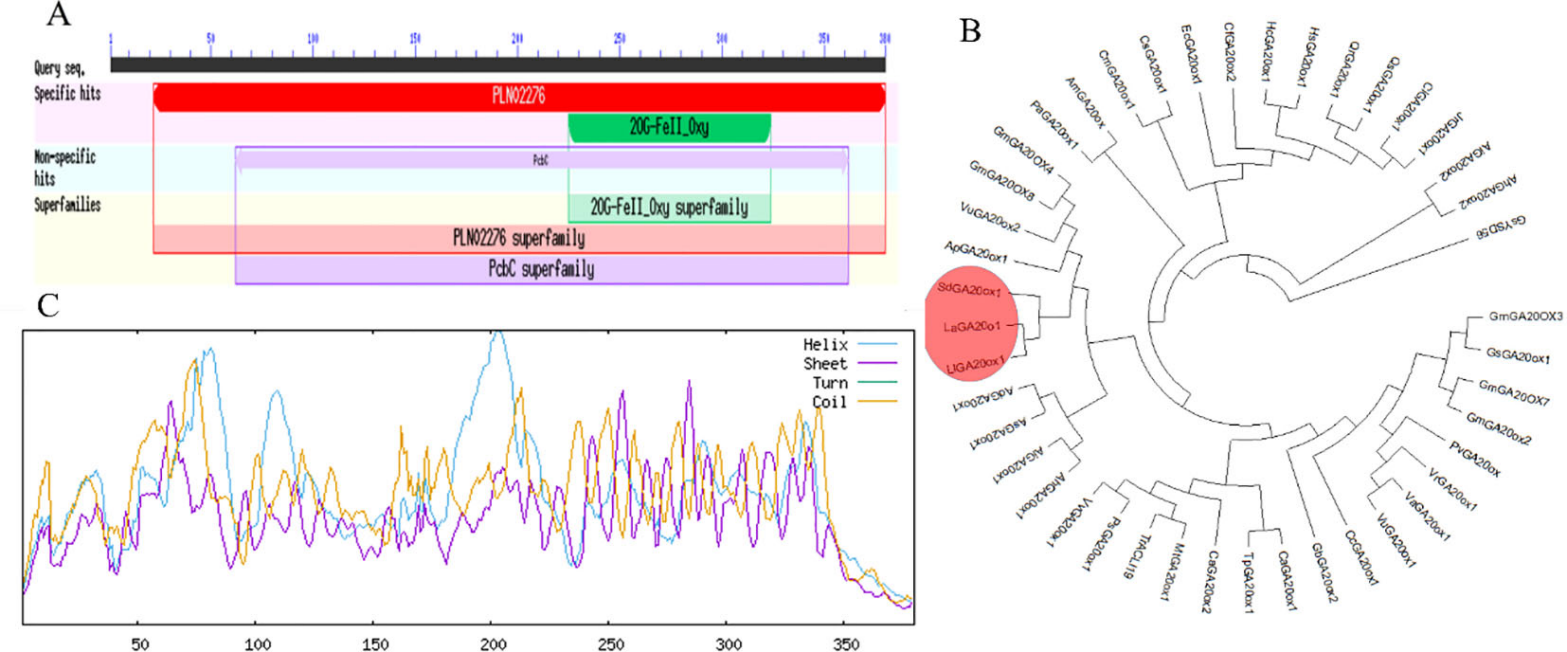
Structural features and phylogenetic analysis of the SdGA20ox1 protein. **(A)** Structure of the SdGA20ox1 protein. **(B)** Phylogenetic tree analysis of the SdGA20ox1 protein based on the amino acid sequence. The red boxes represent other GA20ox1 proteins with the highest identity to the SdGA20ox1 protein. The GenBank accession numbers are as follows: LlGA20ox1: *Lupinus luteus* (MG181995.1), LaGA20o1:*Lupinus angustifolius* (XM_019586625.1), GmGA20OX8:*Glycine max* (XM_003555313.5), GmGA20ox2:*G. max* (EU884445.1), GmGA20OX3:*G. max* (NM_001371636.1), GmGA20OX4:*G. max* (XR_416536.4), GmGA20OX7:*G. max* (NR_163976.2), GsGA20ox1:*Glycine soja* (XM_028324044.1), GsYSD56:*G. soja* (CP154586.1), VuGA20ox2:*Vigna unguiculata* (XM_028067520.1), VuGA20ox1:*V. umbellate* (XM_047326684.1), MtGA20ox1:*Medicago truncatula* (XM_003592315.4), PvGA20ox:*Phaseolus vulgaris* (U70530.1), VaGA20ox1:*Vigna angularis* (XM_017583418.2), VrGA20ox1:*Vigna radiata* var. radiata (XM_014649777.2), CaGA20ox2:*Cicer arietinum* (XM_004496578.3), CaGA20ox1:*C. arietinum* (XM_004513601.3), CcGA20ox1:*Cajanus cajan* (XM_020351662.2), AhGA20ox1:*Arachis hypogaea* (XM_025823797.2), AhGA20ox2:*A. hypogaea* (XM_025800912.2), AiGA20ox1:*Arachis ipaensis* (XM_016321927.2), AiGA20ox2:*A. ipaensis* (XM_016348678.2), AsGA20ox1:*Arachis stenosperma* (XM_057879779.1), ApGA20ox1:*Abrus precatorius* (XM_027511274.1), PaGA20ox1:*Prosopis alba* (XM_028900508.1), AdGA20ox1:*Arachis duranensis* (XM_016086148.3), PsGA20ox1:*Pisum sativum* (XM_051035601.1), VvGA20ox1:*Vicia villosa* (XM_058883955.1), AmGA20ox:*Acacia mangium* (EU252106.1), GbGA20ox2:*Gastrolobium bilobum* (XM_061494988.1), TpGA20ox1:*Trifolium pratense* (XM_045962335.1), QrGA20ox1:*Quercus robur* (XM_050397600.1), QsGA20ox1:*Quercus suber* (XM_024053726.2), CiGA20ox1:*Carya illinoinensis* (XM_043114810.1), JrGA20ox1:*Juglans regia* (XM_018972764.2), CfGA20ox2:*Cornus florida* (XM_059811028.1), CmGA20ox1:*Cucumis melo* (XM_008469033.3), CsGA20ox1:*Cucumis sativus* (XM_004143598.3), HcGA20ox1:*Hibiscus cannabinus* (KY399834.1), EcGA20ox1:*Erigeron canadensis* (XM_043758862.1), HsGA20ox1:*Hibiscus syriacus* (XM_039177100.1), TrACLI19:*Trifolium repens* (CP125836.1). **(C)** secondary structures comparison of the SdGA20ox1 protein. In the figure, Helix: Alpha helix (Hh), Sheet: Extended strand (Ee), Coil: Random coil (Cc), Turn: Beta turn (Tt).

Based on the *S. davidii* transcriptome data (SRA315988) previously obtained by our group, we isolated the full-length cDNA of *SdGA20ox1* (GenBank accession number ON229923) from *S. davidii* plant height mutants using reverse transcriptase-polymerase chain reaction (RT-PCR). The full-length cDNA contained a coding sequence (CDS) of 1143 bp (**Supplementary Figure S1**). The primary structure of the *SdGA20ox1* protein was deduced to contain 380 amino acids, with a predicted molecular mass of 43.16 kDa and an isoelectric point (pI) of 5.96. According to the National Center for Biotechnology Information (NCBI), the *SdGA20ox1* protein contains a conserved 2OG-FeII_Oxy structural domain. Using the NCBI Conserved Domain database, we identified that the protein includes a 2OG-FeII_Oxy structural domain spanning amino acids 225–324, classifying *SdGA20ox1* as part of the 2OG-FeII_Oxy protein superfamily (**Figure 1A**).

To further characterize the *SdGA20ox1* gene within the GA20oxs family, we performed a phylogenetic analysis of the amino acid sequences of GA20oxs from various plant species. The phylogenetic tree revealed that the amino acid sequences of GA20oxs from *S. davidii*, *Lupinus luteus*, and *Lupinus angustifolius* clustered together, indicating a higher homology of *SdGA20ox1* with *LaGA20ox1* and *LlGA20ox1* proteins (**Figure 1B**). The *SdGA20ox1* protein showed the highest identity with *LaGA20ox1* from *Lupinus angustifolius* and *LlGA20ox1* from *Lupinus luteus* (**Figure 1C**). Additionally, The *SdGA20ox1* protein contains three types of helical structures, with alpha helices accounting for 33.68%, irregular coils accounting for 52.11%, and extended chains accounting for 14.21%, which indicated that its main components are α-helices and irregular coils (**Figure 1C**). Based on these findings, we named this gene *SdGA20ox1*.

### 3.2 Expression patterns of *SdGA20ox1* across tissue types and in response to exogenous plant hormone treatment

**Figure 2:**
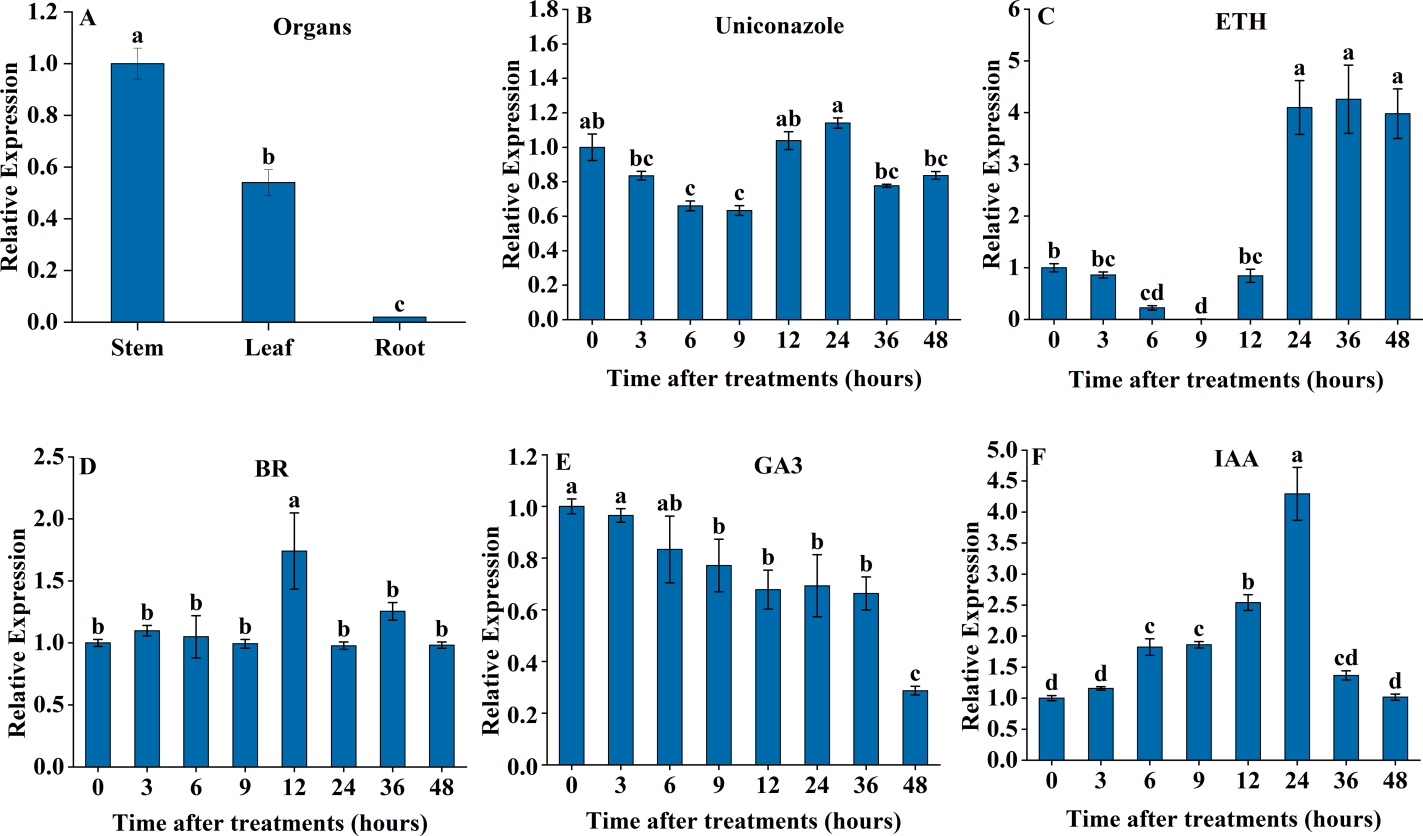
qRT-PCR was used to determine the expression pattern of the SdGA20ox1 gene in adult *S. davidii* mutants. **(A)** Expression of *SdGA20ox1* in stem, leaf, and root. **(B)** Relative expression under 10 μM uniconazole treatment conditions. **(C)** Relative expression under 10 μM ETH treatment conditions. **(D)** Relative expression under 5 μM BR treatment conditions. **(E)** Relative expression under 10 μM GA3 treatment conditions. **(F)** Relative expression after 10 μM IAA treatment conditions. For each treatment, the expression level at 0 h was set as 1.0, and each value represents three replicate experiments. Values are shown as mean ± standard error (SE), and different lowercase letters indicate significant differences (P < 0.05).

The quantitative real-time fluorescence PCR (qRT-PCR) analysis results showed significant differences in *SdGA20ox1* expression across various parts of *S. davidii*, influenced by exogenous phytohormones (**Figure 2**). The gene was significantly more expressed in leaves and stems than in roots (*P* < 0.05), with the highest expression in stems (**Figure 2A**). The expression of *SdGA20ox1* decreased significantly after exogenous spraying of the gibberellin synthesis inhibitor uniconazole; however, it began to increase again after 12 hours, reaching a level not significantly different from 0 hours (**Figure 2B**). Following exogenous ETH treatment, the expression of *SdGA20ox1* showed a trend of decreasing and then increasing, and it was significantly higher than at 0 hours after 24 hours of treatment (**Figure 2C**). After exogenous BR treatment, *SdGA20ox1* expression peaked at 12 hours, then decreased, but remained not significantly different from pre-treatment levels (**Figure 2D**). After exogenous spraying of GA3, *SdGA20ox1* expression continued to decline, eventually decreasing significantly at 48 hours (**Figure 2E**). Conversely, following exogenous spraying of IAA, *SdGA20ox1* expression first increased and then decreased, peaking at 24 hours, which was significantly higher than at other treatment times (**Figure 2F**).

### 3.3 Subcellular localization of *SdGA20ox1*

**Figure 3:**
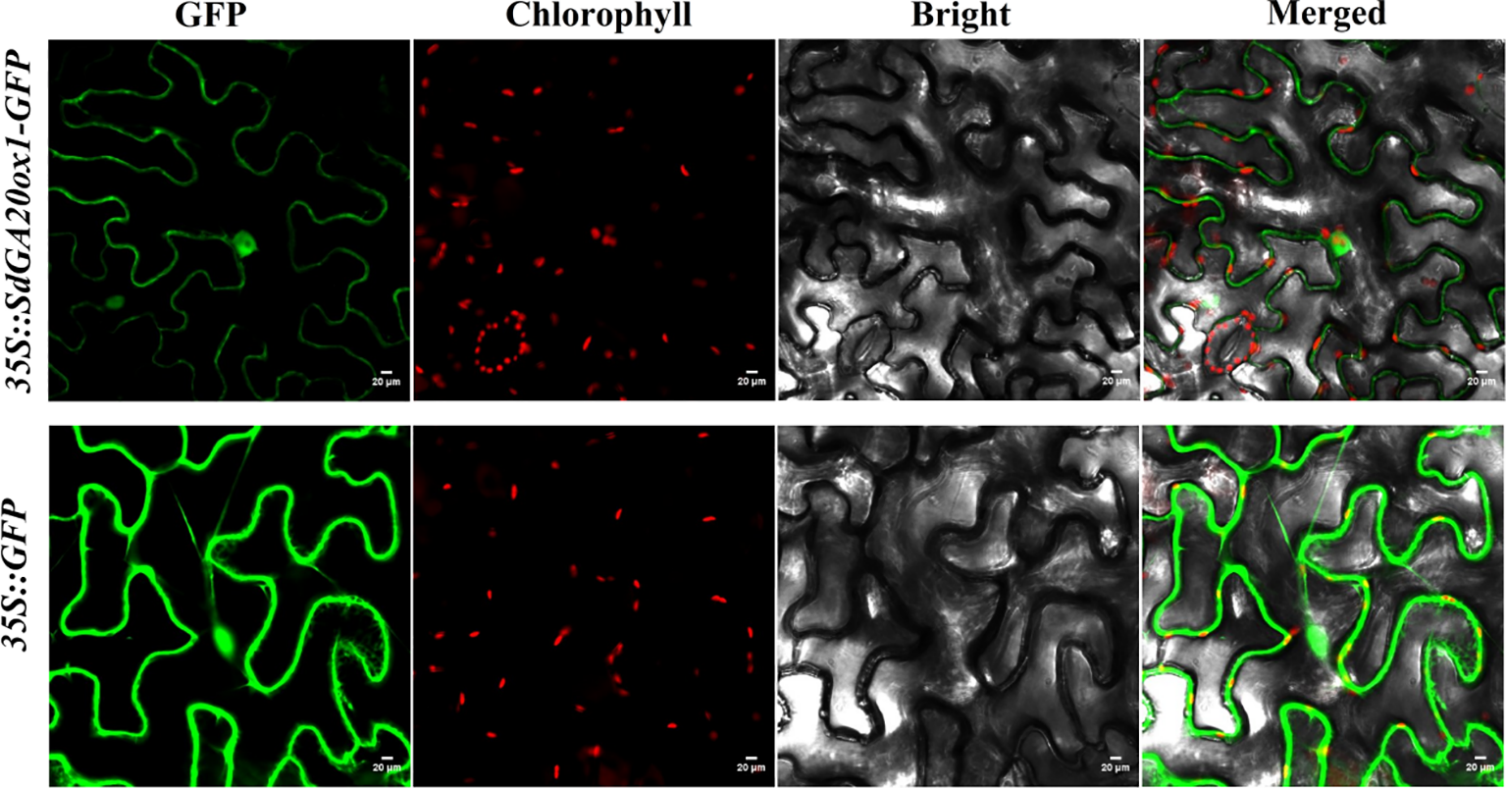
Subcellular localization of *SdGA20ox1*. GFP, green fluorescent protein; chlorophyll, chloroplast channel; bright, bright field; merged, GFP + chlorophyll + bright are shown. Scale bars = 20 μm.

The open reading frame (ORF) of *SdGA20ox1* was fused to the N-terminal of the GFP protein in the CaMV35S promoter-driven pBWA(V)HS-SdGA20ox1-Glosgfp vector to construct the 35S::SdGA20ox1 vector, which was transiently transformed into tobacco leaves. Microscopic observation revealed that SdGA20ox1-GFP was distributed in the nucleus and cytoplasm, with weaker expression in the cell membrane and more concentration in the nucleus compared to the distribution of the GFP control (**Figure 3**).

### 3.4 Phenotype of the *SdGA20ox1* transgenic arabidopsis plants

**Figure 4:**
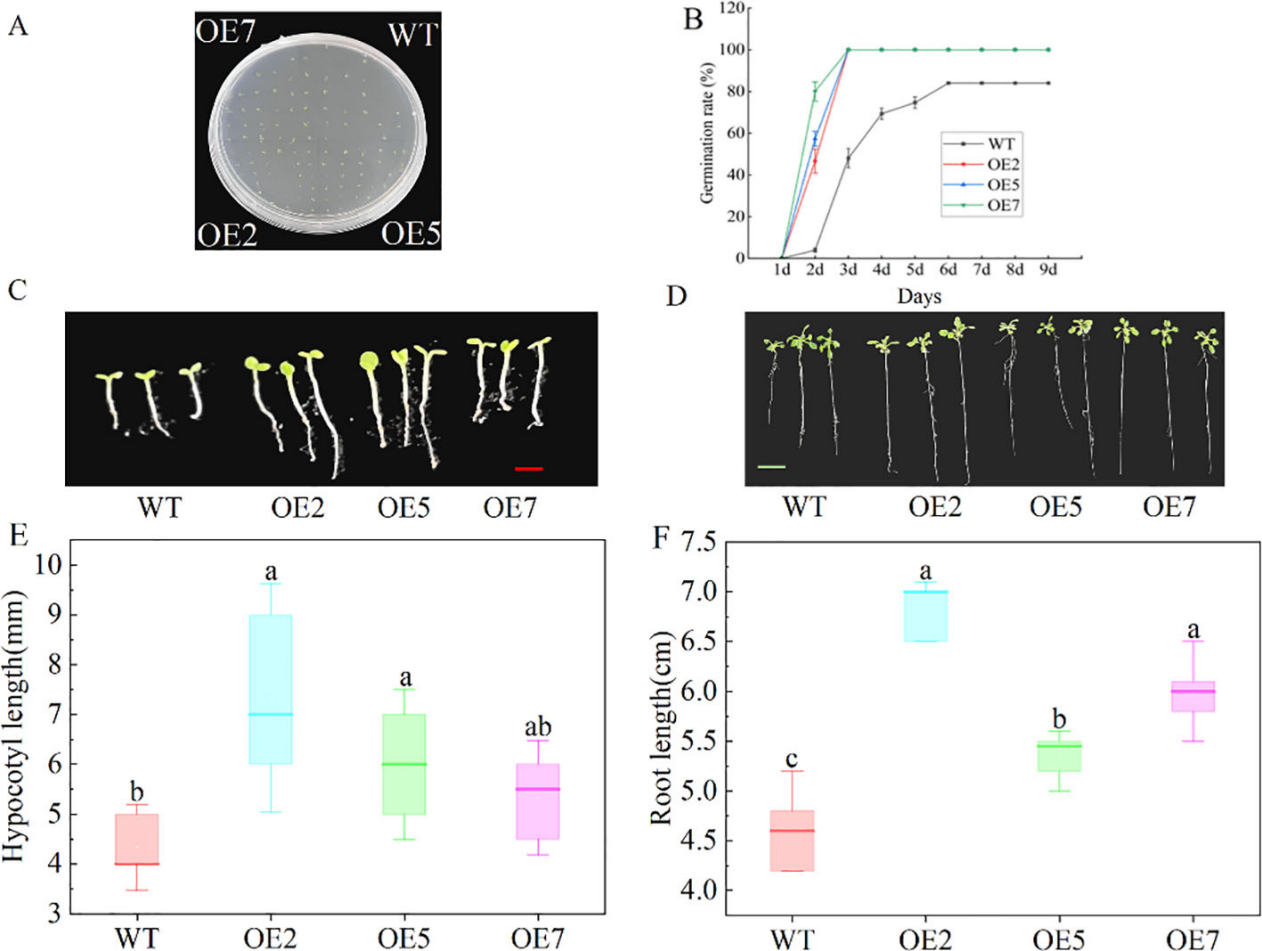
Effect of overexpression of *SdGA20ox1* on seed germination and seedling growth of Arabidopsis. **(A, B)** Arabidopsis germination assay on half-strength MS medium (WT as control), using a total of 30 seeds per treatment and counting germination numbers from day 1 for 9 days. **(C, E)** Hypocotyl length of Arabidopsis seedlings after 7 days of growth on half-strength MS medium. **(D, F)** Root length of Arabidopsis seedlings after 14 days of growth on half-strength MS medium. WT: Col-0 wild-type Arabidopsis control; OE2, OE5, and OE7, separate overexpression lines. Each value represents three replicate experiments. Values are shown as mean ± standard error (SE), and different lowercase letters indicate significant differences (P < 0.05). The red bar in C: 0.5 mm, the green bar in D: 1.25cm.

PCR amplification of target fragments in Arabidopsis resistant plants showed consistency with the positive control band, but the target fragment was not detected in wild-type Arabidopsis (**Supplementary Figure S2**), indicating the successful acquisition of transgenic Arabidopsis. The T2 generation homozygous OE2, OE5, and OE7 lines were further screened for subsequent experiments. During seed germination, the germination rate of seeds from transgenic lines overexpressing (OE) *SdGA20ox1* was significantly higher than that of the wild type (WT), with the germination time also earlier (**Figures 4A, B**). On the second day, the transgenic Arabidopsis seeds germinated in large quantities, with the germination rate of OE7 close to 80%, while the WT lines had a germination rate of less than 20%, significantly lower than that of the transgenic lines (*P* < 0.05).

Compared to the WT, transgenic Arabidopsis seeds sown on half-strength MS agar medium exhibited significantly faster vegetative growth (**Figures 4C, D**). After 7 days of growth on half-strength MS medium, the hypocotyl length of transgenic Arabidopsis seedlings was significantly greater than that of the WT (**Figures 4C, E**), and after 14 days, the root length of transgenic Arabidopsis was significantly greater than that of the WT (**Figure 4F**), indicating that overexpression of *SdGA20ox1* resulted in increased hypocotyl and root lengths in Arabidopsis seedlings.

**Figure 5:**
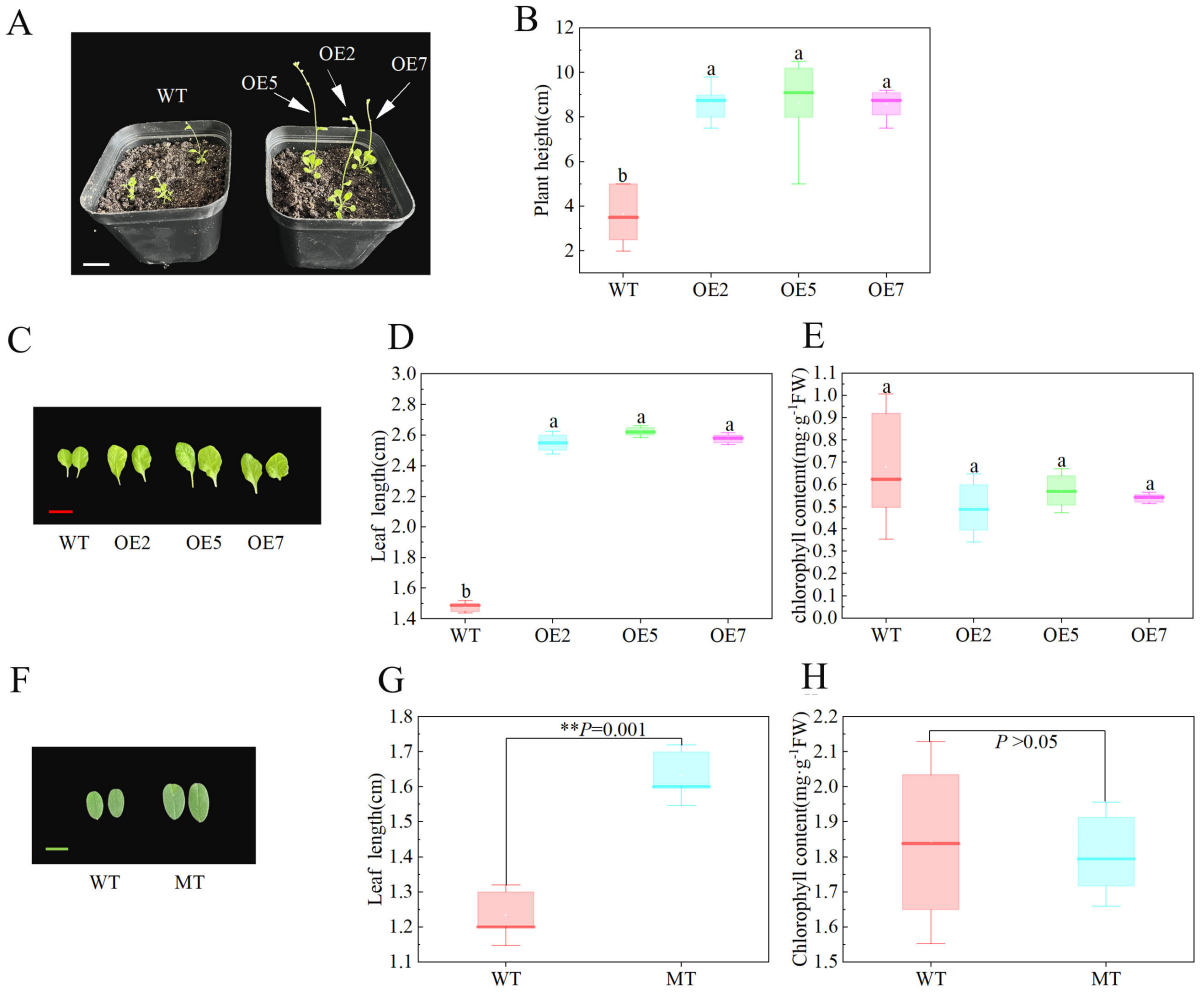
Flowering, leaf, and plant height observations of *SdGA20ox1* overexpression in Arabidopsis. **(A, B)** Flowering stage and plant height observation of Arabidopsis plants at the appearance of the first bud, WT: Col-0 wild-type Arabidopsis control; OE2, OE5, and OE7, separate overexpression lines. **(C–E)**: Leaf length and leaf chlorophyll content measurement of Arabidopsis plants after 45 days of growth, WT: Col-0 wild-type Arabidopsis control; OE2, OE5, and OE7, separate overexpression lines. **(F–H)**: Leaf length and chlorophyll content measurements of 3-month-old *S. davidii* plants, WT: wild-type *S. davidii*, MT: *S. davidii* plant height mutant. Each value represents three replicate experiments. Values are shown as mean ± standard error (SE), different lowercase letters indicate significant differences (P < 0.05), and for **(G, H)**, statistical significance between WT and MT was evaluated by t-tests: **P < 0.01. The white bar in A: 2cm, the red bar in C: 2cm, the green bar in E: 1cm.

**Figure 6:**
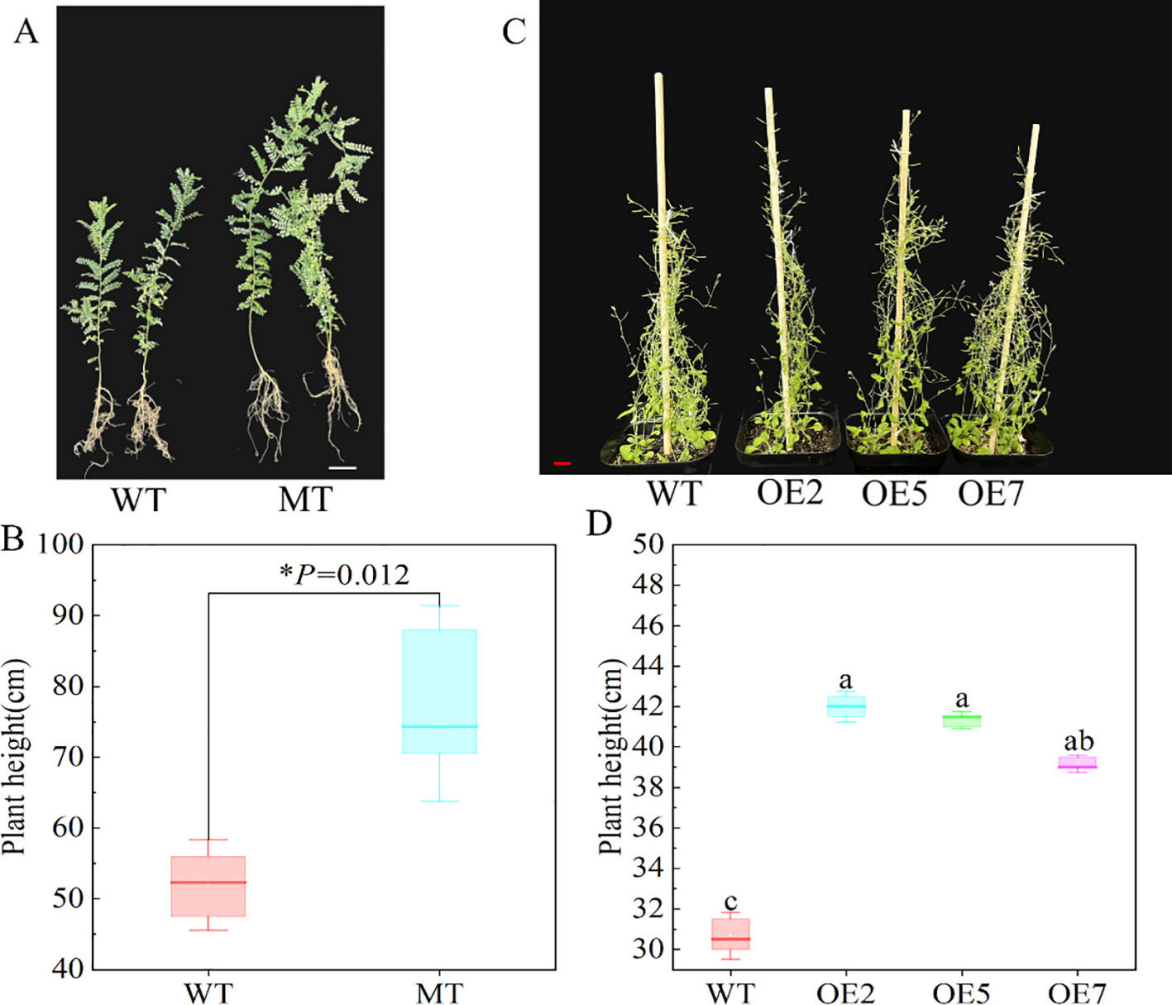
Observation of plant height in Arabidopsis and *S. davidii*. **(A, B)** 3-month-old *S. davidii* plant height, WT: wild type, MT: *S. davidii* plant height mutant. **(C, D)** Arabidopsis plant height after 45 days of growth, WT: Col-0 wild-type Arabidopsis control; OE2, OE5, and OE7, separate overexpression lines. Each value represents three replicate experiments, and values are shown as mean ± standard error (SE), with different lowercase letters indicating significant differences (P < 0.05), and for B, statistical significance between WT and MT was evaluated by t-tests: *P < 0.05. The white bar in A: 1cm, the red bar in C: 2cm.

After transplanting plants grown in half-strength medium for 14 days into nutrient soil, we observed the time of first bud appearance in both WT and transgenic Arabidopsis, along with their plant heights at this time. The experiment showed that transgenic Arabidopsis flowered earlier, with the first bud appearing about 5 days earlier than in the WT (**Figure 5A**). Additionally, when the first bud appeared, the plant height of the transgenic Arabidopsis was significantly higher than that of the WT (*P* < 0.05) (**Figure 5B**). We also measured the leaf size and leaf chlorophyll content of 3-month-old *S. davidii* plant height mutants and Arabidopsis after 45 days of growth. The results showed that the leaf length and plant height of the *S. davidii* plant height mutant were significantly higher than those of the WT *S. davidii* (**Figures 5F, G**, **6A, B**), and the leaf length of transgenic Arabidopsis was also significantly higher than that of the WT (**Figures 5C, D**). However, the chlorophyll content of the leaves in both the *S. davidii* plant height mutant and transgenic Arabidopsis was not significantly different from the WT (P > 0.05), although it was lower (**Figures 5E, H**). After 45 days of growth in nutrient soil, the plant height of transgenic Arabidopsis was significantly higher (*P* < 0.05) than that of WT Arabidopsis across the three overexpression lines (OE2, OE5, and OE7) (**Figures 6C, D**). The plant heights of the OE2, OE5, and OE7 lines increased by 37.0%, 34.8%, and 27.7%, respectively, compared to WT Arabidopsis.

### 3.5 Effect of *SdGA20ox1* overexpression on endogenous GA contents in arabidopsis

**Figure 7:**
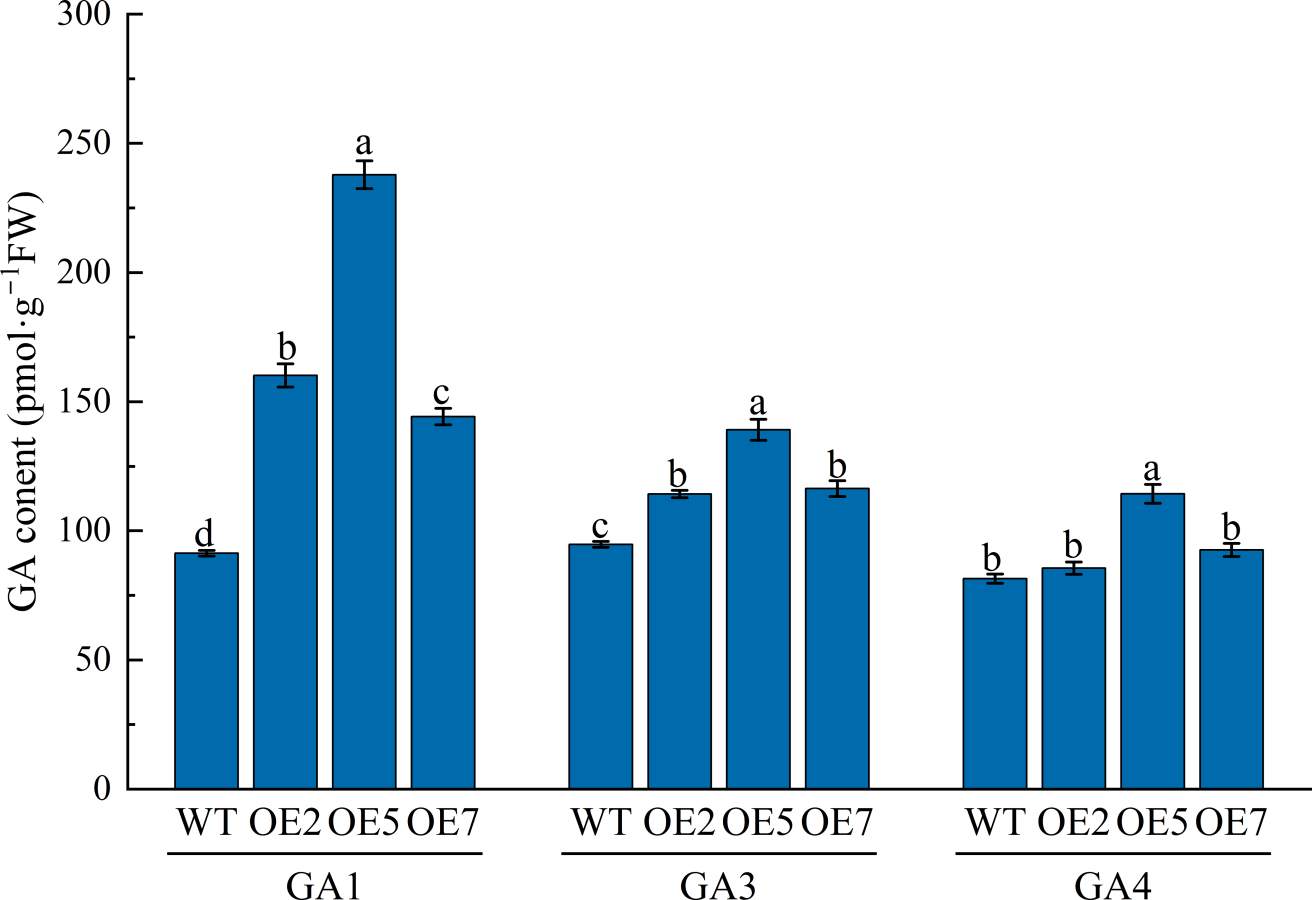
Analysis of endogenous active GA content in WT and transgenic Arabidopsis at 40 days of growth. WT: Col-0 wild-type Arabidopsis control; OE2, OE5, and OE7, separate overexpression lines. Each value represents three replicate experiments, and values are shown as mean ± standard error (SE), with different lowercase letters indicating significant differences (P < 0.05).

We measured the biologically active GA1, GA3, and GA4 contents in young transgenic Arabidopsis (OE2, OE5, and OE7) stems grown for 40 days (**Figure 7**). The results showed that GA1 and GA3 contents in transgenic plants were significantly higher than in WT (*P* < 0.05), approximately 1.8-fold, 2.6-fold, and 1.6-fold for GA1 and 1.2-fold, 1.5-fold, and 1.2-fold for GA3, respectively. While GA4 content in transgenic plants was higher than in WT, only OE5 showed a significant difference (*P* < 0.05), being about 1.4-fold higher than WT.

### 3.6 Construction of cDNA library and library screening for *S. davidii* plant height mutants

We constructed a cDNA library with a capacity of 5.84 × 10^7^ CFU/ml and a total number of clones of 1.168 × 10⁸ CFU (**Supplementary Figure S3**). To analyze the band distribution structure of the constructed library, 24 randomly selected clones were subjected to colony PCR using T7-F/AD-R primers (**Supplementary Table S1**.). PCR electrophoresis results showed that the average size of the library fragments was 1000 bp (**Supplementary Figure S4**).

**Figure 8:**
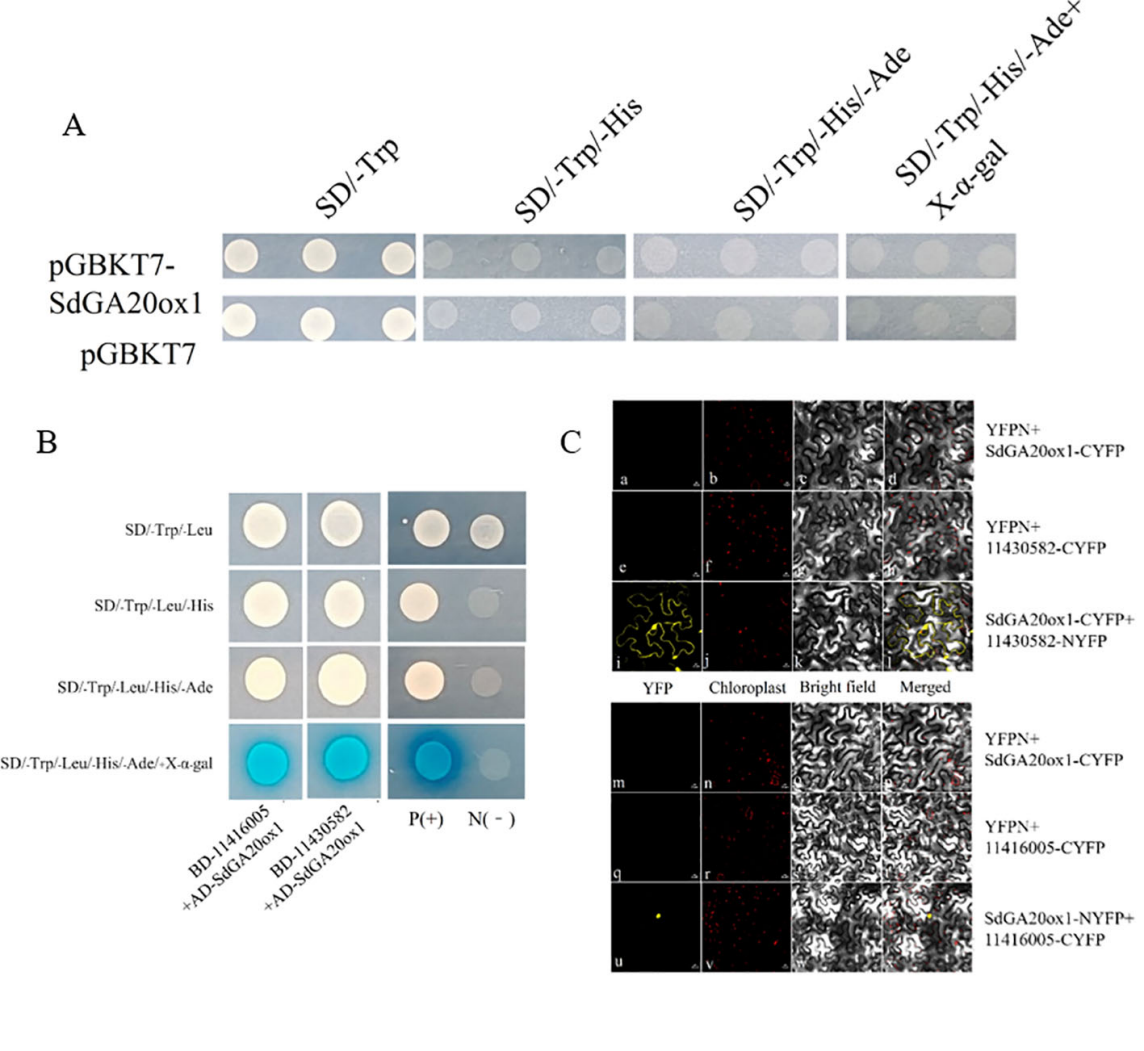
Validation of interaction protein of SdGA20ox1. **(A)** Analysis of the autoactivation and toxicity of pGBKT7-SdGA20ox1. pGBKT7 is the negative control. SD medium without tryptophan (SD/-Trp), without tryptophan and histidine (SD/-Trp/-His), without tryptophan, histidine, and adenine (SD/-Trp/-His/-Ade), and without tryptophan, histidine, adenine, and containing X-α-gal (SD/-Trp/-His/-Ade/+X-α-gal). **(B)** Co-transformed yeast cells were dropped onto SD medium without tryptophan and leucine (SD/-Trp/-Leu), without tryptophan, leucine, and histidine (SD/-Trp/-Leu/-His), without tryptophan, leucine, histidine, and adenine (SD/-Trp/-Leu/-His/-Ade), and without tryptophan, leucine, histidine, adenine, and containing 200 mg/mL X-α-gal (SD/-Trp/-Leu/-His/-Ade/+X-α-gal). pGADT7-largeT+pGBKT7-p53 was used as a positive control (P+) and pGADT7-largeT+pGBKT7-laminC was used as a negative control (N-). **(C)** For the assessment of PpSm35s-SdGA20ox1 and PpSm35s-11430582, BiFC analysis of the interaction between PpSm35s-11416005. (a, e, i, m, q, u) YFP fluorescence images; (b, f, j, n, r, v) chloroplast images; (c, g, k, o, s, w) bright-field images; and (d, h, l, p, t, x) fluorescence images merged with their respective bright-field images.

We used Y2H to screen for interacting proteins. Autoactivation analysis showed that pGBKT7-SdGA20ox1 had no autoactivation (**Figure 8A**). The AH109 yeast strain containing the pGBKT7-SdGA20ox1 decoy plasmid was used as a receptor to prepare the sensory state, into which the library plasmid pGADT7-S. davidii cDNA was subsequently transferred and plated on SD-TLH plates. Screening on SD-TLH plates yielded 192 positive yeast clones (**Supplementary Figures S5**, **S6**). We then amplified and DNA sequenced the 192 positive clones from yeast cells. BLAST analysis of the sequences in the GenBank database resulted in 28 reciprocal protein gene sequences (**Supplementary Figure S7**; **Supplementary Table S2**).

### 3.7 Interaction test of *SdGA20ox1* interacting proteins in plants

To further validate the possible interaction of candidate proteins with *SdGA20ox1*, we selected two genes from the 28 candidate genes with predicted functions related to plant growth and development: *rbcS* (11416005) and *EIN4* (11430582). We constructed pGADT7-11416005 and pGADT7-11430582 vectors for point-to-point yeast transformation with pGBKT7-SdGA20ox1. The results showed that the positive control could grow on SD/-Trp/-Leu, SD/-Trp/-Leu/-His, SD/-Trp/-Leu/-His/-Ade, and SD/-Trp/-Leu/-His/-Ade/+X-α-gal plates, and displayed a blue color on SD/-Trp/-Leu/-His/-Ade/+X-α-gal plates. The negative control could grow only on SD/-Trp/-Leu plates. In the experimental group, the growth of pGBKT7-SdGA20ox1 + pGADT7-11416005 and pGBKT7-SdGA20ox1 + pGADT7-11430582 was consistent with the positive control, indicating that both *11416005* and *11430582* physically interact with *SdGA20ox1* in yeast (**Figure 8B**).

*11416005* is involved in plant photosynthesis, while *11430582* may function in the early stages of the ethylene signaling pathway. We used the BiFC assay to confirm the interaction between *SdGA20ox1* and *11416005* and *11430582*. A distinct YFP yellow fluorescent signal was observed in tobacco leaf pulp protoplast cells after cotransformation with N-terminal YFPN fusion *11430582* and C-terminal YFPC fusion *SdGA20ox1*, as well as after cotransformation with N-terminal YFPN fusion *SdGA20ox1* and C-terminal YFPC fusion *11416005*. However, no YFP signal was detected in the negative controls (YFPN and YFPC-SdGA20ox1, YFPN and 11430582-YFPC, YFPN and YFPC-SdGA20ox1, and YFPN and 11416005-YFPC) (**Figure 8C**). These results suggest that the *SdGA20ox1* protein can physically interact with *11416005* and *11430582* in plants, and the three may affect endogenous gibberellin production through these interactions.

## 4 Discussion

*Sophora davidii* is a common diploid cross-pollination plant in the subtropical karst region of southwest China, characterized by a complex genetic background. The absence of complete genome sequencing has made gene mining and isolation more challenging compared to other crops (Sun et al., 2022). As an excellent plant for soil and water conservation, with high nutritional and medicinal values, optimizing the genetic architecture of *S. davidii* is crucial for promoting local ecological animal husbandry and improving stone desertification soil. In this study, through a preliminary transcriptomic analysis of an *S. davidii* mutant, we cloned a gibberellin 20-oxidase homologue gene, *SdGA20ox1*, and predicted that the secondary structure of this protein is predominantly α-helix. By comparing the *SdGA20ox1* protein with GA20ox proteins from legumes such as *Lupinus luteus*, *Lupinus angustifolius*, and *Cicer arietinum*, we found that they share the same conserved structural domain (Griggs et al., 1991), indicating the evolutionary conservation of GA20ox proteins in legumes. The subcellular localization of *SdGA20ox1* to the nucleus and cytoplasm is similar to that of *CoGA20ox1* (Wang et al., 2023). These results provide a reference for a more comprehensive study of gibberellins and the growth and development of *S. davidii*.

qRT-PCR results showed that *SdGA20ox1* was expressed in the roots, stems, and leaves of *S. davidii*, with the highest expression in the stem, indicating that *SdGA20ox1* expression in plant stems is a major factor determining the height of *S. davidii* plants. This finding aligns with the study by Wang et al. (2018), which reported significantly higher expression of *CrGA20ox1* in *Camellia reticulata* stems compared to leaves. So, Gibberellin regulation may involve feedback from other plant hormones or growth regulators during plant development (Zhao et al., 2022b; Luo et al., 2015). In this study, the expression of *SdGA20ox1* initially decreased upon exogenous application of the plant growth retardant uniconazole to *S. davidii*, then began to increase again. This may be due to uniconazole inhibiting the activity of upstream genes of *GA20ox1*, reducing the reaction substrate of the *GA20ox1* gene, and consequently decreasing its expression initially. A previous study also reported a decrease in *GA20ox* expression after 24 hours of uniconazole treatment in *Landoltia punctata*. However, no effect on *HaGA20ox1* gene expression was observed when sunflower was treated with gibberellin inhibitors (Carzoli et al., 2009). Conversely, *SdGA20ox1* expression continued to decrease and was significantly lower than the control 6 hours after exogenous GA3 application. This is consistent with studies showing a continuous decrease in *GA20ox* gene expression after gibberellin treatment in sugarcane, eventually lower than the untreated control (Wu et al., 2009). These results suggest that exogenous gibberellins significantly inhibit *GA20ox* gene expression in different plants.

Exogenous *ETH* application significantly increased *SdGA20ox1* expression in *S. davidii* from 24 to 48 hours, implying that *ETH* may positively regulate *SdGA20ox1* expression. There are fewer studies on the relationship between ETH and *GA20oxs*, but *ETH* is known to play an important role in plant growth and development as a unique plant hormone (Dugardeyn and van der Straeten, 2008). Our study showed that *SdGA20ox1* expression increased and then decreased after exogenous application of BR and IAA, with significant differences in the periods of highest expression. Exogenous BR application was able to upregulate *GA20ox* gene expression, as demonstrated by exogenous BR spraying in Arabidopsis mutants, which upregulated *AtGA20ox1* expression (Ozga and Reinecke, 2003). Previous research found that *AtGA20ox1* gene expression increased after IAA treatment of Arabidopsis seedlings under light conditions (Goda et al., 2004), and another study showed that low IAA concentration (30 mg/L) treatments transiently increased *PsGA20ox1* gene expression levels in pea (*Pisum sativum*) pericarp (Ngo et al., 2002).

In this study, Arabidopsis seeds overexpressing *SdGA20ox1* showed higher and earlier germination rates compared to wild-type (WT) seeds. Similar results were found by Gao and Ayele (2014), who demonstrated that *GA20ox* plays a crucial role in promoting wheat (*Triticum aestivum*) seed germination. This suggests that *SdGA20ox1* overexpression increases endogenous gibberellin content in seeds, enhancing germination speed and rate. Our study also found that *SdGA20ox1* overexpression significantly promoted hypocotyl and root elongation in Arabidopsis seedlings, increased plant height and leaf length, and led to earlier flowering in transgenic plants. These findings are consistent with previous research indicating that overexpression of *GA20oxs* results in hypocotyl and root elongation, increased plant height, and early flowering in transgenic Arabidopsis (Coles et al., 1999; Eriksson et al., 2000; Wang et al., 2023; Wu et al., 2023).

Additionally, the *S. davidii* mutant and transgenic Arabidopsis overexpressing *SdGA20ox1* exhibited lighter leaf colors and lower chlorophyll content, though the difference in chlorophyll content was not significant. In contrast, overexpression of *CoGA20ox1* and *EguGA20ox1* significantly decreased chlorophyll content in Arabidopsis (Wang et al., 2023; Wu et al., 2023). These findings suggest that the regulation of leaf color and chlorophyll content by GA differs among plant species. In our study, the active GA content (GA1, GA3, and GA4) was significantly higher in transgenic plants than in WT, with GA1 showing the largest increase. This suggests that *SdGA20ox1* may promote growth by increasing endogenous active GA content, particularly GA1, thereby altering plant phenotypes. Similarly, GA1 plays a crucial role in controlling branch elongation in peach (*Prunus persica*) and aspen (*Populus tremula*) (Zhang et al., 2022; Israelsson et al., 2005), while GA4 is the key regulatory factor in controlling shoot elongation in *Camellia reticulata* (Wang et al., 2018) and *Oryza sativa* (Qin et al., 2013). These research results demonstrate that the main biological activity GA varies at different stages of tissue development in some plant species (Hu et al., 2017).

The bioactive GA1 and GA4 are synthesized from GA12 through two parallel pathways (early-13-hydroxylation and non-13-hydroxylation) by two kinds of 2-oxoglutarate dependent dioxygenases in the cytoplasm, GA20 oxidase (*GA20ox*) and GA3 oxidase (*GA3ox*). The promotion of shoot and internode elongation is a common phenomenon in GA-overproducing or GA-treated plants. Overexpression of the SdGA20-oxidase gene increases levels of intermediates for bioactive GAs, promoting cell elongation and/or cell division, thereby increasing shoot heights. This effect has been observed in *GA20ox* transgenic plants of various species, such as *Zea mays* (Liu et al., 2024), *Camellia reticulata* (Wang et al., 2018), hybrid poplar (*Populus tremula* × *P. alba*) (Fladung, 2022), *Camellia oleifera* (Wang et al., 2023), and rice (*Oryza sativa*) (Qin et al., 2013). Conversely, semi-dwarf or dwarf phenotypes have been observed in transgenic plants overexpressing the GA20 oxidase gene from *Torenia fournieri* (Otani et al., 2014) and *Cucurbita maxima* (Radi et al., 2006), similar to GA20 oxidase-deficient rice mutants. This variation in phenotypes suggests different functions of GA20 oxidase genes in regulating plant height in different species, possibly due to feedback regulation of GA20 oxidase gene expression by bioactive GA.

The construction of an efficient yeast two-hybrid (Y2H) library supports protein interaction research and potential gene screening, providing indispensable conditions for an in-depth understanding of protein function and revealing mechanisms of action (Wang et al., 2018). Our homogenized full-length cDNA library of *S. davidii*, with a titer of 5.84 × 10_7_ cfu/ml and an average insertion fragment length of 1000 bp, meets the requirements of high-quality cDNA libraries (Clarke and Carbon, 1976) and is reliable for subsequent yeast hybridization screening. Our analysis indicates that *SdGA20ox1* protein can physically interact with *rbcS* (11416005) and *EIN4* (11430582) proteins in yeast and tobacco cells.

The *RbcS* gene encodes a small subunit of the key enzyme RuBisCo (Ribulose-1,5-bisphosphate carboxylase/oxygenase), involved in photosynthetic carbon assimilation (Paritosh et al., 2013). The expression level of the *RbcS* gene correlates with Rubisco efficiency, ultimately affecting photosynthetic efficiency (Zhuang et al., 2020). This has been widely confirmed in pea (*Pisum sativum*) (Kuhlemeier et al, 1987), *Zea mays* (Silverthorne and Tobin, 1990), poplar (*Populus deltoides* × *P. euramericana* cv ‘Nanlin895’) (Wang et al., 2013), cotton (*Gossypium hirsutum*) (Paritosh et al., 2013), and tomato (*Solanum lycopersicum*) (Zhuang et al., 2020). A significant increase in *RbcS* gene expression levels was observed in tomatoes, which may help improve the photosynthetic efficiency and nutritional growth of tomatoes. At the same time, *GA20* gene expression levels were similar to that of *RbcS* gene, significantly increased by three times, promoting tomato growth, and both jointly regulate tomato growth (Zhuang et al., 2020). In this study, both yeast two-hybrid and BiFC assays showed that SdGA20ox1 protein can interact with RbcS protein, potentially leading to increased photosynthesis and gibberellin synthesis, promoting better nutritional growth of *S. davidii*, similar to findings in tomatoes (Zhuang et al., 2020).

Ethylene plays crucial roles in various processes of plant growth, development, and stress responses (Gallie, 2015). Ethylene is perceived through its binding to receptors located in the endoplasmic reticulum (Shakeel et al., 2013). Once ethylene binds to the receptors, various responses are observed in seed germination, stem cell division, cell elongation and differentiation, root hair growth, fruit ripening, senescence, abscission, and responses to abiotic stresses, confirmed in Arabidopsis, tomato (*Solanum lycopersicum*), maize (*Zea mays*), and other plants (Hua and Meyerowitz, 1998; Tieman et al., 2000; Gallie and Young, 2004). EIN/EILs play an important role in the ethylene signaling pathway and can also cross regulate plant traits with other plant hormone signals. For example, in rice, *EIN3* binds to the promoters of SK1 and SK2 to promote the expression of both, activating the gibberellin response required for stem elongation and regulating stem elongation in rice (Ayano et al., 2014). *EIN4* is also one of the ethylene receptors. In this study, results indicate that SdGA20ox1 protein may interact with EIN4 protein, affecting ethylene response and regulating plant stem growth. However, further experimental verification is needed to confirm the specific pathway of this interaction.

## 5 Conclusion

Overexpression of *SdGA20ox1* in Arabidopsis resulted in reduced chlorophyll accumulation in plant leaves, earlier flowering, elongation of seedling hypocotyl and root lengths, and increased plant height. These findings suggest that *SdGA20ox1* could be a potential candidate gene for selecting new dwarf varieties of *S. davidii* using gene editing technology. By constructing a nuclear yeast library, we predicted and screened two proteins that interact with *SdGA20ox1*. Further yeast two-hybrid (Y2H) and bimolecular fluorescence complementation (BiFC) assays demonstrated that SdGA20ox1 interacts with the ethylene receptor EIN4 and the RBCS proteins, indicating its involvement in the regulation of plant growth and development.

## Data Availability

The datasets presented in this study can be found in online repositories. The names of the repository/repositories and accession number(s) can be found in the article/**Supplementary Material.**
