# Supplementary material for: Characterization of the gibberellic oxidase gene SdGA20ox1 in Sophora davidii (Franch.) skeels and interaction protein screening
Source: Front Plant Sci. 2024 Oct 16;15:1478854. doi: 10.3389/fpls.2024.1478854 (PMC11521860; doi:10.3389/fpls.2024.1478854)
Supplement: Supplementary file 1 [file DataSheet1.docx]

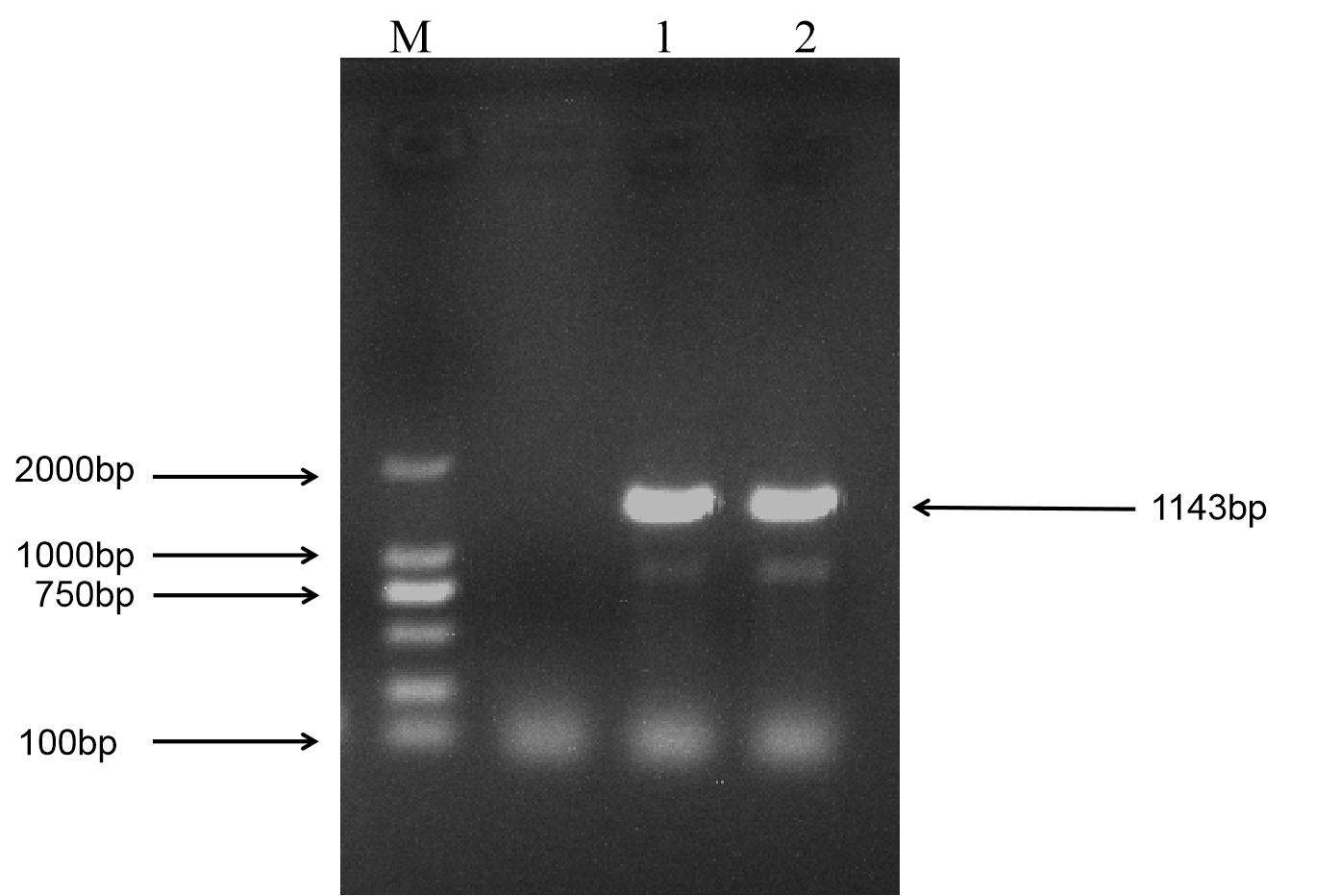


**Figure S1 Cloning of *SdGA20ox1***

Note: M: 2000 DNA Marker. 1, 2: *SdGA20ox1* gene banding.


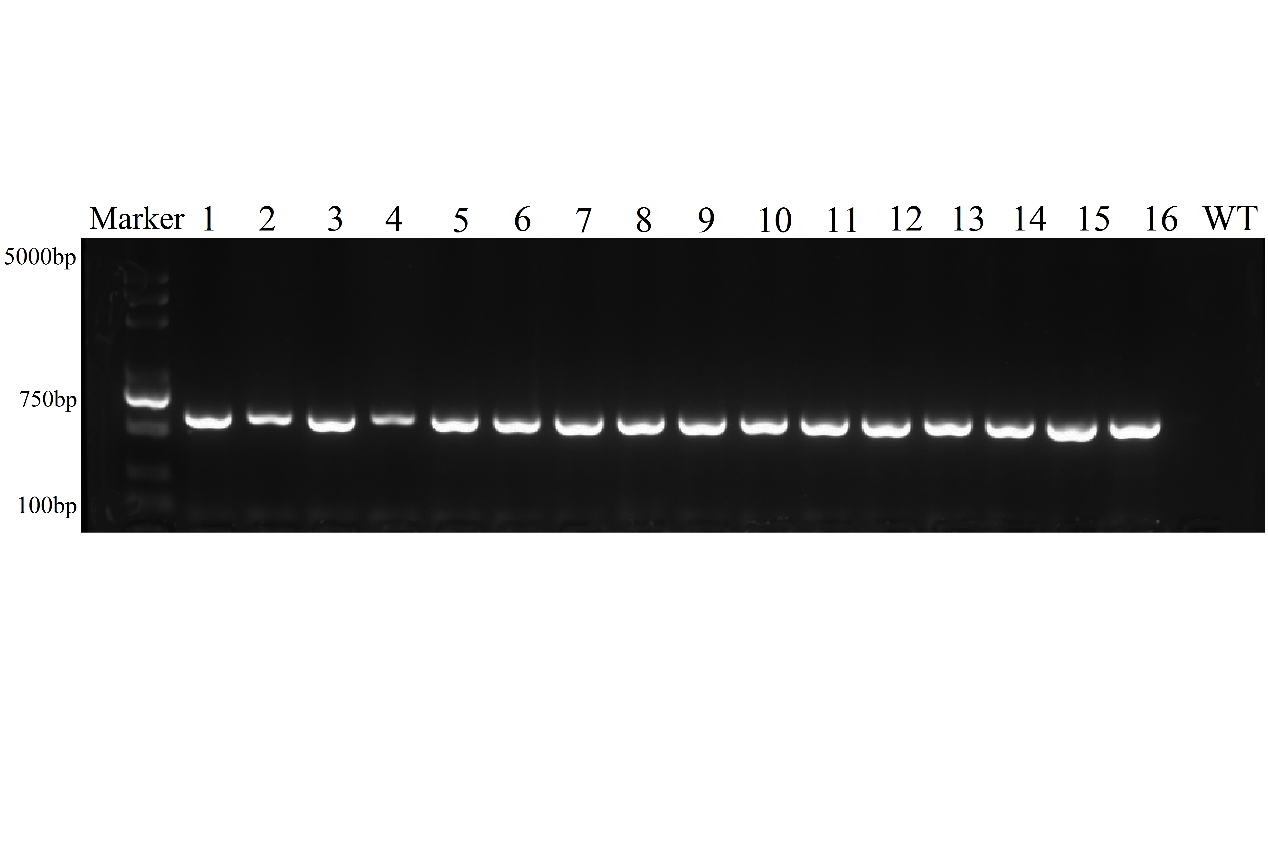


**Figure S2 Transgenic Arabidopsis thaliana PCR assay**

Note: 1-16: Selected resistant Arabidopsis; WT: wild-type Arabidopsis.


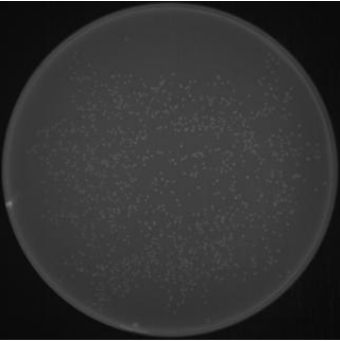


**Figure S3 *E. coli* colony count**

Note: Clone number 584, library capacity (CFU/ml) = number of clones/coating volume × dilution factor, total number of clones (CFU)=library capacity × total volume of bacterial solution, resulting in a library capacity of 5.84 × 10 ⁷ CFU/ml and a total number of clones of 1.168 × 10 ⁸ CFU.


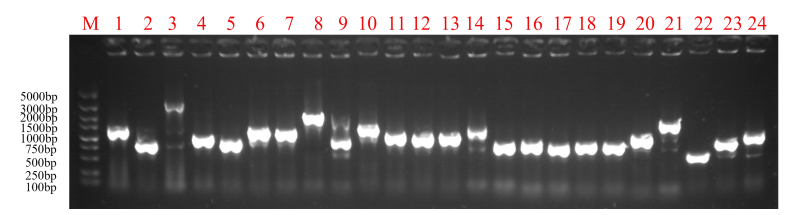


**Figure S4 Library quality identification**

Note: M：Maker; 1-24：24 randomly selected clones


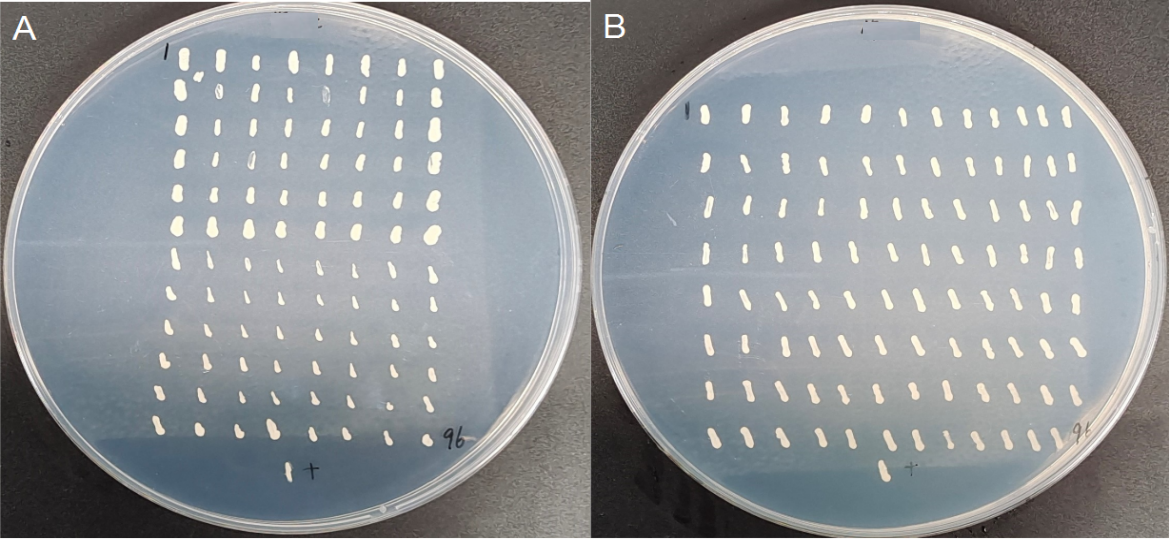


**Figure S5 Screening of positive yeast clones in SD-TLH plates**

Note: A, 1-96 samples in the first culture dish; B, 1-96 samples in the second culture dish. (+)：positive control pGADT7-largeT+ pGBKT7-p53


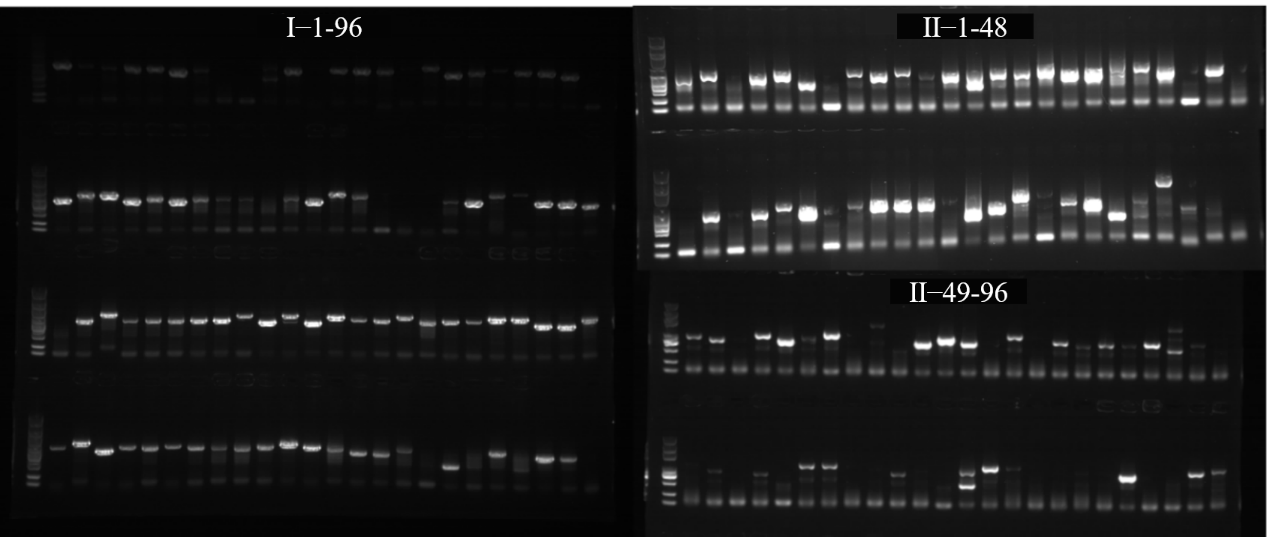


**Figure S6 PCR identification of positive yeast colonies**


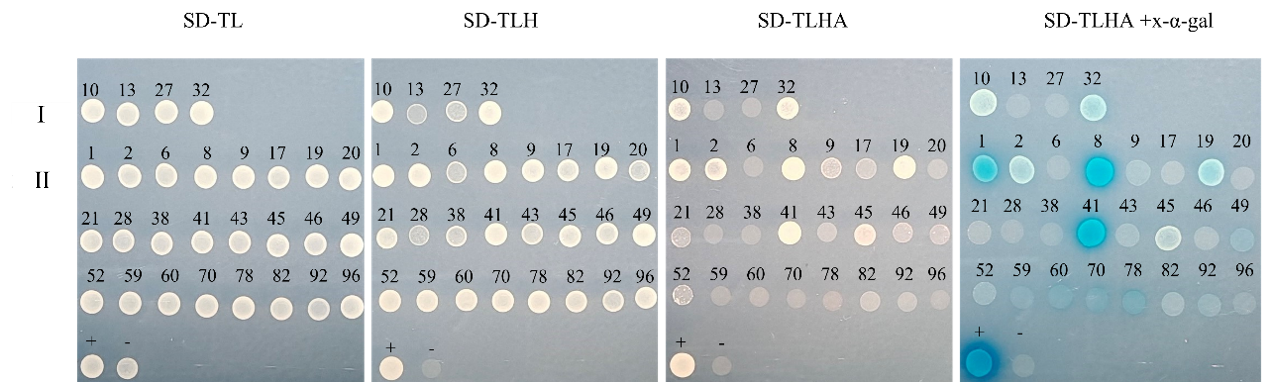


**Figure S7 Rotation verification results**

Note: (+) positive control pGADT7-largeT + pGBKT7-p53; (-) negative control pGADT7-largeT + pGBKT7-laminC
